# Identification of the LOX Gene Family in Peanut and Functional Characterization of *AhLOX29* in Drought Tolerance

**DOI:** 10.3389/fpls.2022.832785

**Published:** 2022-03-09

**Authors:** Yifei Mou, Quanxi Sun, Cuiling Yuan, Xiaobo Zhao, Juan Wang, Caixia Yan, Chunjuan Li, Shihua Shan

**Affiliations:** Shandong Peanut Research Institute, Qingdao, China

**Keywords:** peanut, lipoxygenase, gene family, expression pattern, drought stress

## Abstract

Lipoxygenases (LOXs) are a gene family of nonheme iron-containing dioxygenases that play important roles in plant development and defense responses. To date, a comprehensive analysis of *LOX* genes and their biological functions in response to abiotic stresses in peanut has not been performed. In this study, a total of 72 putative *LOX* genes were identified in cultivated (*Arachis hypogaea*) and wild-type peanut (*Arachis duranensis* and *Arachis ipaensis*) and classified into three subfamilies: 9-LOX, type I 13-LOX and type II 13-LOX. The gene structures and protein motifs of these peanut LOX genes were highly conserved among most LOXs. We found that the chromosomal distribution of peanut LOXs was not random and that gene duplication played a crucial role in the expansion of the LOX gene family. Cis-acting elements related to development, hormones, and biotic and abiotic stresses were identified in the promoters of peanut *LOX* genes. The expression patterns of peanut *LOX* genes were tissue-specific and stress-inducible. Quantitative real-time PCR results further confirmed that peanut *LOX* gene expression could be induced by drought, salt, methyl jasmonate and abscisic acid treatments, and these genes exhibited diverse expression patterns. Furthermore, overexpression of *AhLOX29* in *Arabidopsis* enhanced the resistance to drought stress. Compared with wide-type, *AhLOX29*-overexpressing plants showed significantly decreased malondialdehyde contents, as well as increased chlorophyll degradation, proline accumulation and superoxide dismutase activity, suggesting that the transgenic plants exhibit strengthened capacity to scavenge reactive oxygen species and prevent membrane damage. This systematic study provides valuable information about the functional characteristics of AhLOXs in the regulation of abiotic stress responses of peanut.

## Introduction

Peanut is one of the most important economic crops producing healthy oil and high-quality protein for global human diets (Bertioli et al., 2016). The cultivated peanut (AABB genome) originated from two diploid progenitor species: *Arachis duranensis* and *Arachis ipaensis* (Bertioli et al., 2016; Zhuang et al., 2019). However, substantial peanut crop losses occur worldwide each year due to a variety of abiotic and biotic stresses, such as drought, salt, herbivorous insects and viruses. In plants, the synthesis of oxylipins and their derivatives, such as jasmonic acid (JA), green leaf volatiles, divinyl ethers and traumatic acid, is catalyzed by LOXs in the LOX pathway (Silke and Baldwin, 2010; Christensen et al., 2015). These oxylipins have been evidenced to be involved in plant responses to various stresses (Porta and Rocha-Sosa, 2002; Zhou et al., 2009).

Lipoxygenases are nonheme and iron-containing dioxygenases that compose multiple subfamilies in plants, fungi and animals (Brash, 1999). On the basis of specific LOX localization during the process of oxygenating substrates, including linoleic acid, α-linolenic acid (α-LeA), and arachidonic acid, plant LOXs have been divided into two classes: 9-LOXs and 13-LOXs. Furthermore, 13-LOXs can be classified into two subfamilies: type I 13-LOXs and type II 13-LOXs. Type I 13-LOX genes exhibit high sequence similarity (75%) and have no transit peptides, while type II 13-LOX genes exhibit low similarity with each other (up to 35%) and possess a chloroplast transit peptide (Veldink et al., 1977; Brash, 1999). The LOX pathway in plants contains four major metabolic routes: the peroxygenase pathway, the allene oxide synthase pathway, the hydroperoxide lyase pathway and the divinyl ether synthase pathway (Feussner and Wasternack, 2002; Porta and Rocha-Sosa, 2002). In the LOX metabolic pathway, LOXs catalyze polyunsaturated fatty acids, such as LA, α-LeA and arachidonic acid, to produce either 13S- or 9S-hydroperoxy derivatives (Feussner and Wasternack, 2002). These hydroperoxides are then metabolized *via* several secondary reactions and converted into diverse oxylipins. The different oxylipins generated by these metabolic routes participate in plant development (tendril coiling, cell death, etc.) and defense responses (responses to wounds, pathogens, fungi, herbivory, and other stresses; Reymond et al., 2000). In addition, LOXs have been proved to be key factors regulating the growth, lipid metabolism, seed storage and vigor, maturation and senescence, as well as stress resistance in soybean, rice, maize, and peanut (Carrera et al., 2007; Ying and Yz, 2020). More recent studies have focused on the functions of plant LOXs, especially with regard to growth, development, and stress responses.

Due to the rapid development of structural and functional genomics, many *LOX* genes were identified in a wide range of plants, and their functions in development (Liu and Chervin, 1997; Kolomiets et al., 2001; Yan et al., 2017) and defense responses (Heitz et al., 1997; Hui et al., 2016a) have also been well studied. A total of six LOXs (*AtLOX1* to *AtLOX6*) were characterized in *Arabidopsis* (Umate, 2011). With the applications of high-performance liquid chromatography and gas chromatography–mass spectrometry analysis, *AtLOX1* and *AtLOX5* were classified as 9-LOXs, and *AtLOX2*, *AtLOX3*, *AtLOX4* and *AtLOX6* were classified as 13-LOXs (Bannenberg et al., 2009). *AtLOX1* and *AtLOX5* are reportedly involved in lateral root development and defense against pathogens (Tamara et al., 2007). Oxylipins produced by the 9-lipoxygenase pathway in *Arabidopsis* regulate lateral root development and defense responses through a specific signaling cascade (Tamara et al., 2007). *AtLOX3* and *AtLOX4* were proved not only to be essential for male fertility but also to be involved in global proliferation (Caldelari et al., 2011). Besides, *Arabidopsis lox3 lox4* double mutants are male sterile, and the double mutation results in defective global proliferative arrest; these genes perform distinct functions in resistance to plant-parasitic nematodes (Ozalvo et al., 2014). *AtLOX2* and *AtLOX6* participate in the JA biosynthesis pathway, and can be induced by different stresses (Bell et al., 1995; Grebner et al., 2013). In addition, rice *OsHI-LOX*, maize *ZmLOX10* and tobacco *NaLOX3* mediate herbivore-induced defense, and they are involved in the JA biosynthesis (Zhou et al., 2009; Rayko and Baldwin, 2010; Christensen et al., 2013). In maize, the *LOX8* localizes to chloroplasts and participates in the wound-induced JA biosynthesis pathway, during which the production of *LOX10*-derived oxylipins is essential (Christensen et al., 2013). Furthermore, the *ZmLOX10* localizes to organelles and acts to modulate both direct and indirect defenses against herbivores (Christensen et al., 2013).

The LOX gene family has been systematically studied in several plants, such as *Arabidopsis*, tomato, and maize (Bannenberg et al., 2009; Mariutto, 2011; Ogunola et al., 2017), but data for peanut was sparse. In peanut, scientists casted most attention on the potential roles of *LOX* genes in resistance to *Aspergillus flavus*. The phylogenetic relationships and molecular functions (*Aspergillus flavus* infection) of wild-type peanut *LOX* genes have been investigated (Hui et al., 2016b). A body of evidence has demonstrated that *LOX* and its products 9S- and 13S-hydroperoxy fatty acids (9S- and 13S- HPODE) play a significant role in the *Aspergillus*/seed interaction (Burow et al., 2000; Willis, 2005; Müller et al., 2014; Korani et al., 2018; Khan et al., 2020). However, the genome-wide identification and functional characterization of the LOX gene family under abiotic stresses in cultivated peanut has not been conducted. The availability of peanut genome annotation information made it possible to systematically and comprehensively characterize the LOX gene family. Here, 72 putative peanut LOX genes were identified by genome-wide searches of the wild-type and cultivated peanut genomes. The phylogenetic relationships, gene structures, conserved protein motifs and structures, subcellular localization, chromosome localization, gene duplication as well as cis-acting elements of *LOX* genes were characterized in peanut. To investigate the expression patterns of peanut LOXs in different tissues and under various stress challenges, the RNA-seq results were integratedly studied. Moreover, the expression patterns of five LOXs from three subfamilies in response to two stress treatments (drought and salt) and two hormone treatments (MeJA and ABA) were determined. These results will contribute to the selection of candidate genes for further characterization of peanut LOX genes which function in growth, development and defenses against various abiotic stresses.

## Materials and Methods

### Identification of LOXs in Peanut

The protein sequences of the allotetraploid *Arachis hypogaea* (AABB) and its two wild diploid progenitor species *A. duranensis* (AA) and *A. ipaensis* (BB; Milligan et al., 1998) were obtained from the PeanutBase database.^1^ The GmLOX amino acid sequences were downloaded at https://phytozome.jgi.doe.gov/pz/portal.html, and the MtLOX protein sequences were downloaded at http://jcvi.org/medicago/display.php?pa-geName=General&section=Download.

A local BLAST search was performed to identify putative peanut *LOX* genes. First, the HMM profile of lipoxygenase (accession: PF00305) was obtained from PFAM,^2^ and the peanut local protein database was downloaded from PeanutBase. The versions of the peanut genomes from PeanutBase used in this study were as follows: Cultivated peanut (Version 2: *A. hypogaea* cv. *Tifrunner*) and *A. ipaensis* (Version 2). The HMM profile was then utilized to identify putative peanut LOXs in the peanut local protein database *via* the hmmsearch tool in HMMER3.0 software. All the putative *LOX* genes were further confirmed to contain LOX domains using the PFAM databases^3^ (Finn et al., 2006), InterPro^4^ (Quevillon et al., 2005) and the NCBI Batch CD-search database^5^ (Marchler-Bauer et al., 2005). In addition, the physical and chemical properties of the putative LOX proteins, including the number of amino acids (NA), molecular weight (Liu et al., 2016) and isoelectric point (theoretical pI), were calculated using the online tool ExPASy^6^ (Gasteiger et al., 1999). Detailed information on these physical and chemical properties of the peanut LOXs was presented in Supplementary Table S1.

### Phylogenetic Analysis

Multiple alignments of LOX proteins from four species, including peanut (*A. duranensis*, *A. ipaensis* and *A. hypogaea*), *Glycine max*, *Medicago truncatula* and *Arabidopsis thaliana*, were conducted using ClustalW. The Gblocks Server^7^ was used to select conserved protein blocks for the above multiple alignment. A maximum likelihood phylogenetic tree with 1,000 bootstrap replicates was constructed *via* Molecular Evolutionary Genetics Analysis (MEGA X) software (Sudhir et al., 2018). The tree was further edited with specific colors indiciating the different subfamilies by EvolView.^8^

### Analysis of Gene Structures, Conserved Motifs and Promoters

The exon–intron structures were visualized with the online tool Gene Structure Display Server (GSDS) using the CDS and genomic sequences of peanut LOXs^9^ (Guo et al., 2007). The conserved motifs of the peanut LOX proteins were identified using Multiple Expectation Maximization for Motif Elicitation (MEME Suite; Timothy et al., 2006).^10^ The promoter sequences (1.5 kb upstream of the peanut LOX transcription start site) were downloaded from PeanutBase, and were used to predict cis-acting regulatory elements with PlantCARE.^11^ Detailed information on the cis-acting elements in each LOX promoter was shown in Supplementary Table S4.

### Prediction of the 3D Structures of LOX Proteins

**Figure 1:**
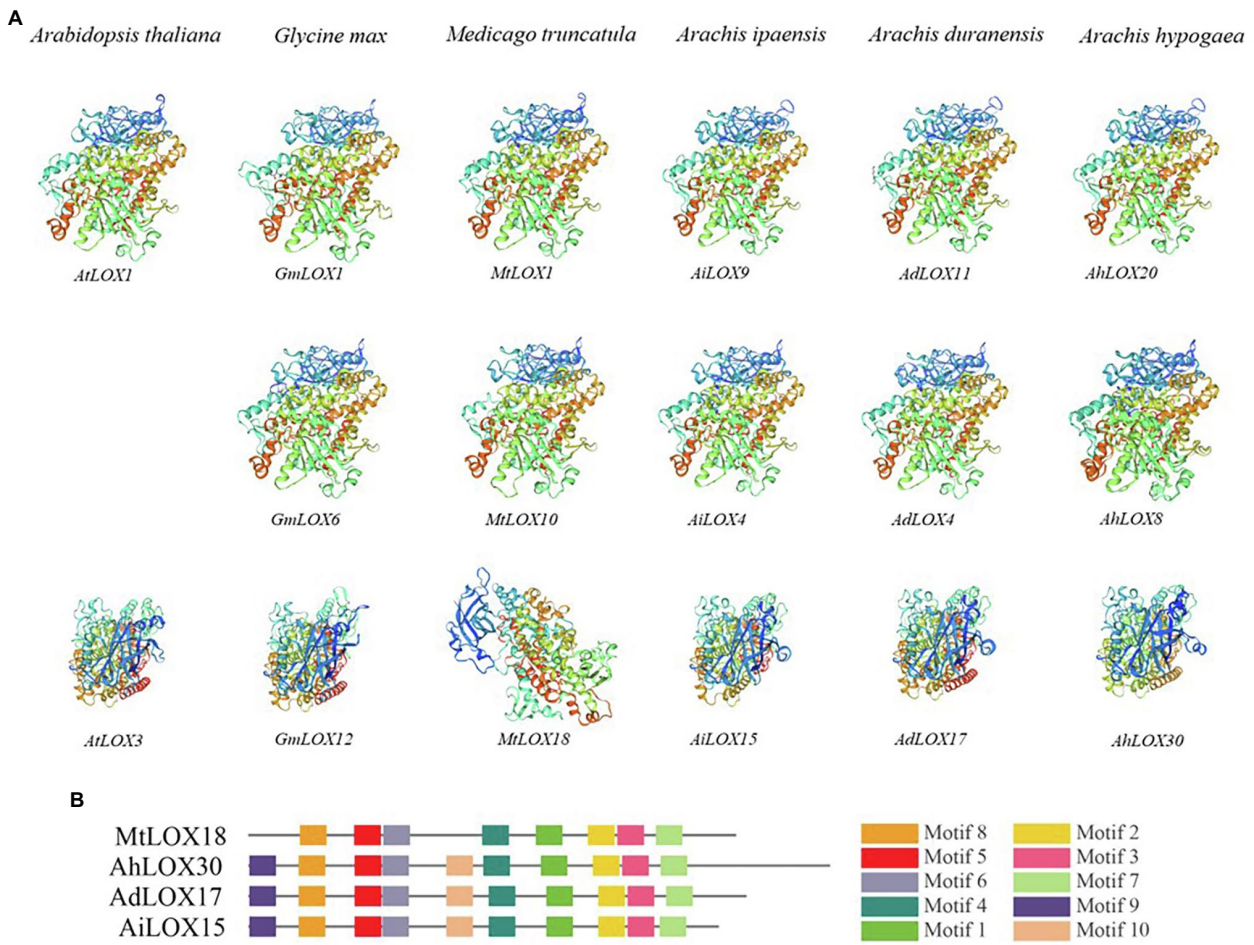
Predicted structures of LOX proteins. The modeled structures of LOX genes from different species are displayed. 9-LOX (AtLOX1, GmLOX1, MtLOX1, AiLOX9, AdLOX11, and AhLOX20), type I 13-LOX (GmLOX6, MtLOX10, AiLOX4, AdLOX4, and AhLOX8) and type II 13-LOX (AtLOX3, GmLOX12, MtLOX18, AiLOX15, AdLOX17, and AhLOX30).

The 3D protein structures of the LOXs were predicted using SWISS-MODEL^12^ (Andrew et al., 2018). We randomly selected genes from each subfamily and from different species for modeling. As shown in Figure 1, 9-LOX (AtLOX1, GmLOX1, MtLOX1, AiLOX9, AdLOX11, and AhLOX20), type I 13-LOX (GmLOX6, MtLOX10, AiLOX4, AdLOX4, and AhLOX8) and type II 13-LOX (AtLOX3, GmLOX12, MtLOX18, AiLOX15, AdLOX17, and AhLOX30) were displayed.

### Chromosomal Location and Gene Duplication

The chromosomal locations of the putative *LOX* genes in peanut were obtained from the PeanutBase database.^13^ Gene duplication events of the peanut *LOX* genes were analyzed (E-value<1e-10) using the Multiple Collinearity Scan toolkit (MCScanX).^14^ The segmental duplicated genes were characterized as homologous genes between different chromosomes. To visualize the segmentally duplicated pairs of peanut *LOX* genes, we draw a map of the chromosomal distribution and duplication of peanut *LOX* genes using Circos software.^15^

### Analysis of the Gene Expression Patterns of Peanut *LOX* Using RNA-Seq Values

To study the expression patterns of peanut *LOX* genes in different tissues and under different stresses, the fragments per kilobase of transcript per million fragments (FPKM) values of 22 tissues were obtained from the PeanutBase database (Josh et al., 2016),^16^ and the FPKM values of *LOX* genes in response to drought and salt treatments were obtained from our previous work. The *LOX* accession number and FPKM values of different tissues and under different stresses were provided in Supplementary Tables S6 and S7. A total of 72 *LOX* gene expression data points were log2-transformed and visualized by Heml software (Wankun et al., 2014).

### Plant Materials and Treatments

Seedlings of the peanut cultivar Huayu71 (a cultivated peanut bred by our team) were grown until the three-leaf stage and used for the gene expression analysis. All the materials were grown in a climate chamber under controlled conditions: 20°C /16 h light and 20°C/8 h dark, with 60% relative humidity. For the drought and salt stresses, the seedlings were immersed in Hoagland liquid medium supplemented with 20% PEG6000, 200 mM NaCl, 100 μM MeJA or 100 μM ABA. The roots of the seedlings were harvested after 0, 6, 12, 24, and 48 h and rapidly frozen in liquid nitrogen. Three biological replicates were performed for the stress analysis in this study.

### RNA Isolation and Quantitative Real-Time PCR Analysis

Total RNA was extracted using the Takara RNA Extraction kit (Code No. 9767, TaKaRa, Dalian) according to the manufacturer’s instructions. First-strand cDNA was synthesized using the Takara PrimeScript RT Reagent kit (Code No. RR037, Takara, Dalian). The quantitative real-time PCR (qRT–PCR) was carried out in a 20 μl reaction system containing 1 μl (100 ng) of cDNA, 0.4 μl of forward primer (10.0 μmol/l), 0.4 μl of reverse primer (10.0 μmol/l), 10 μl of 2 × qPCR mixture (2× Takara TB Premix Ex Taq Mix, No. RR820, Takara, Dalian) and 8.2 μl of RNase-free water using the ABI7500 Fast System (Applied Biosystems, CA, United States). The PCR procedure was set as follows: 95°C for 5 min; 40 cycles of 95°C for 10 s and 60°C for 30 s. The relative expression levels of all the genes were analyzed using 2^–∆∆CT^. The primers specific for the AhLOX genes and actin used in this work were shown in Supplementary Table S8.

### Vector Construction and Transformation Into Arabidopsis

The coding DNA sequence (CDS) of *AhLOX29* was amplified from “Huayu71” cDNA. The 2,754-bp CDS was then ligated between a 35S promoter and the nopaline synthase (Nos) terminator in the pCambia2300EC vector to construct the overexpression vector (35S. *AhLOX29*. Nos). The constructed overexpression plasmid was transformed into *Arabidopsis* according to an established transformation procedure. After being cultured on MS media supplemented with 50 mg/l kanamycin, positive seedlings were obtained from the selective media. Subsequently, the surviving seedlings were transplanted to nutritional soil for further confirmation by PCR using the vector-specific primers: VSP-F and VSP-R (Supplementary Table S8).

### Determination of Drought-Related Physiological Parameters

Drought stress treatment was applied to both WT and transgenic plants while they grew to the seedling stage in a climate chamber. The WT and transgenic plants were grown in the same amount of nutrient soil, and the same amount of water was poured at each watering. The total chlorophyll contents, proline contents, malondialdehyde (MDA) contents and superoxide dismutase (SOD) enzyme activity were measured before drought treatment. The four physiological parameters were measured after water was stopped for 10 days. The total chlorophyll contents in leaves were determined using the chlorophyll assay kit (Comin, CPL-2-G, Suzhou). Leaves of *Arabidopsis* plants were measured for three replicates, and the average values were calculated as total chlorophyll levels. For quantification of the proline contents, MDA contents and SOD enzyme activity, the assay Kit (Comin, PRO-2-Y, MDA-2-Y and SOD-2-W, Suzhou) was used following previously described procedures (Bates et al., 1973; Hao et al., 2013; Yu et al., 2015).

## Results

### Identification of Peanut *LOX* Genes and Phylogenetic Analysis of LOXs From Different Species

To identify *LOX* genes in peanut, the HMM profile of LOX (accession: PF00305) was used to search the peanut local protein database. A total of 89 putative *LOX* genes were identified in peanut. After confirming the presence of the LOX domain using the PFAM, InterPro and NCBI Batch CD-search databases, 72 peanut *LOX* genes were determined to have the whole lipoxygenase domain and were used for further analysis. Based on the analysis of physical and chemical properties, we found that the length of peanut LOX proteins ranged between 509 (AdLOX5) and 1,491 amino acids (AhLOX25), and the molecular weight of LOX proteins ranged from 58.35 kDa (AdLOX5) to 170.64 kDa (AhLOX25). The pI was predicted to range from 4.98 (AhLOX5) to 9.15 (AiLOX17), which indicated that various microenvironments were needed for each LOX protein to perform optimal function.

**Figure 2:**
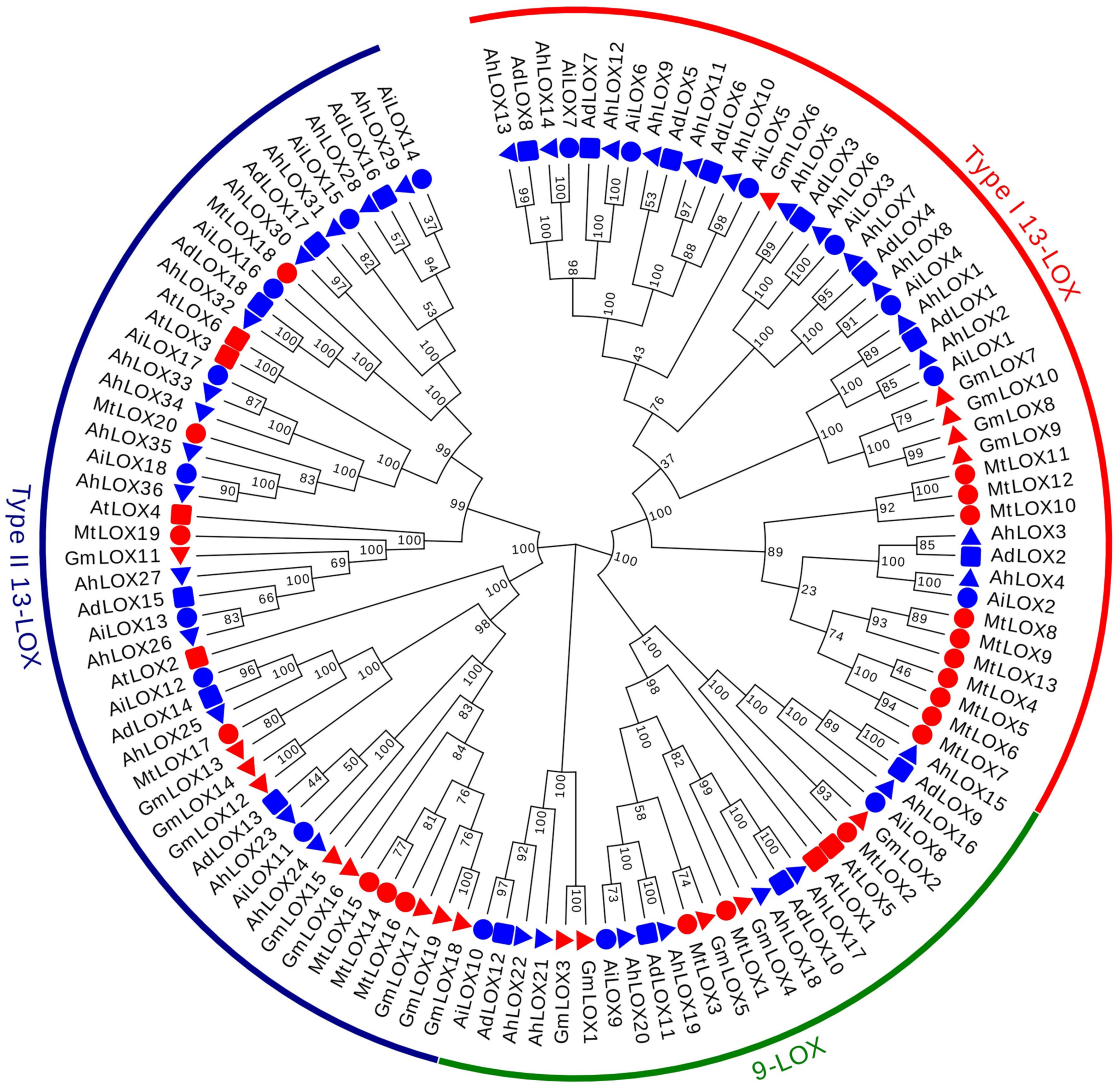
Phylogenetic tree of the peanut LOX gene family. Phylogenetic tree of 117 LOX proteins from peanut (72), *Arabidopsis* (6), *Glycine max* (19) and *Medicago truncatula* (20). MEGA X software was used to construct the maximum-likelihood (ML) phylogenetic tree with 1,000 replications.

The classification and phylogenetics of LOX proteins have been analyzed in several plants, such as *Arabidopsis*, *Glycine max*, and *Medicago truncatula* (Jin et al., 2008; Umate, 2011; Ogunola et al., 2017), helping us to classify the evolutionary relationships of the peanut LOX proteins. Multiple sequence alignments of 117 LOX conserved protein blocks, including 72 peanut LOXs, 6 AtLOXs, 19 GmLOXs and 20 MtLOXs, were performed using ClustalW, and their GenBank accession IDs were presented in Supplementary Table S2. A maximum likelihood phylogenetic tree with 1,000 bootstraps was constructed using the LOX proteins of these four species. On the basis of the phylogenetic relationships and the classifications of LOX proteins from other species, the peanut LOX gene family could be classified into three clades: 9-LOX, type I 13-LOX and type II 13-LOX (Figure 2). As shown in Figure 2, the peanut LOXs were randomly distributed among the LOXs of the other three species. In the 9-LOX subfamily, AdLOX9-12, AiLOX8-10, and AhLOX15-22 are closely related to AtLOX1, AtLOX5, GmLOX1-5, and MtLOX1-3. AdLOX1-8, AiLOX1-7, and AhLOX1-14 are type I 13-LOXs and are closely related to GmLOX6-10 and MtLOX4-13. In the type II 13-LOX clade, AdLOX13-18, AiLOX11-18, and AhLOX23-36 are closely related to AtLOX2-4, AtLOX6, GmLOX11-19, and MtLOX14-20. The bootstrap values for some parts of the phylogenetic tree are low, which may have occurred due to the low similarity of protein sequences.

### Phylogenetic, Gene Structure and Conserved-Domain Analysis of Peanut *LOX* Genes

**Figure 3:**
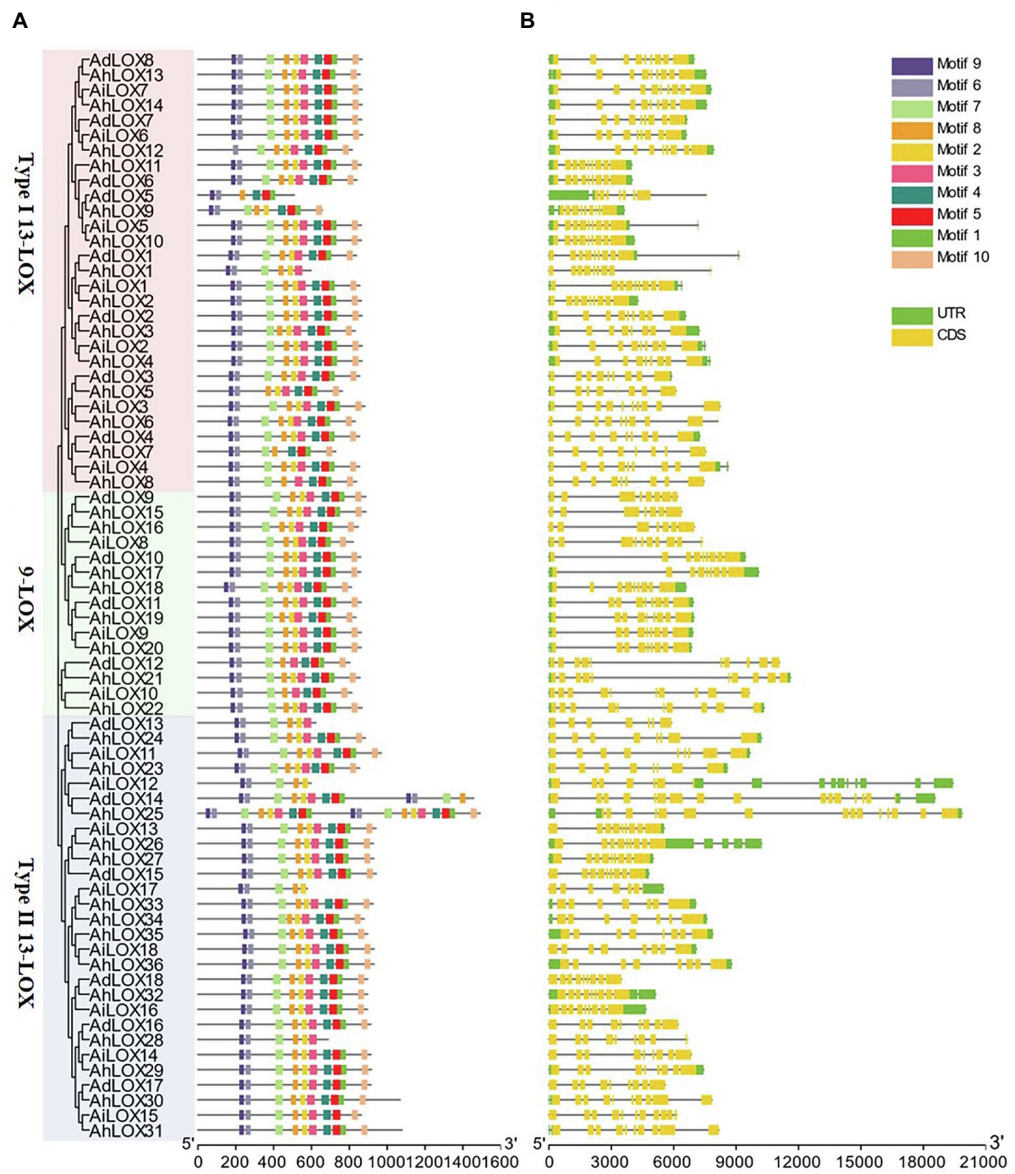
Phylogenetic relationship, gene structure and conserved-motif analysis of the *LOX* genes in peanut. The maximum-likelihood phylogenetic tree was constructed using MEGA X with 1,000 replicates. **(A)** Conserved motifs of *LOX* proteins. Ten conserved motifs are shown in different-colored boxes, and detailed information on the motifs is provided in Supplementary Table S3. **(B)** Exon-intron structures of *LOX* genes. The yellow boxes represent exons, and the gray lines represent introns.

To gain a further understanding of the gene structure and conserved protein motifs of LOX genes, we constructed a maximum likelihood phylogenetic tree with the peanut LOX proteins, and compared the protein motifs (Figure 3A) and exon/intron structures (Figure 3B) of each LOX gene. The exon/intron structures of the LOXs were analyzed using GSDS. As shown in Figure 3B, most LOXs were found to have similar gene structures in terms of both intron numbers (7–9) and exon lengths. However, variations in the intron numbers of some LOXs were observed. The maximum number of introns (17 introns) was observed in AdLOX14. In addition, we found that AhLOX25 had 15 introns while AiLOX15 and AhLOX30-31 contained 10 introns.

The LOX protein motifs were identified by searching the MEME Suite databases to explore the similarity and diversity of motifs within subfamilies. We identified 10 distinct motifs in the peanut LOX proteins, named as motifs 1–10 (Figure 3A and Supplementary Table S3). As expected, all peanut LOX proteins contained highly representative motifs, including 38-residue (motif 1), substrate-binding (motif 3), and oxygen-binding (motif 2) motifs. Among them, motif 1 was essential for the stability of LOX protein, and the AhLOX1, AhLOX28, AdLOX13, AiLOX12, and AiLOX17 lacked this motif, indicating that these genes might exhibit altered enzymatic activity. In addition, motif 5 (except AhLOX1, AhLOX28, AdLOX13, AiLOX12, and AiLOX17), motif 6, motif 7 (except AdLOX5 and AhLOX5), motif 8, motif 9, and motif 10 (except AdLOX5, AhLOX1, AdLOX13, AiLOX12, AiLOX17, and AhLOX28) were conserved in all peanut LOXs. The AhLOX1, AdLOX13, AiLOX12, AiLOX17, and AhLOX28 lacked motif 4. Whether the absence of these motifs confers unique functions to these proteins requires further investigation. In any case, the highly conserved motifs in peanut LOX proteins would contribute to the functional analysis of the peanut LOX proteins.

### Analysis of the Predicted Structures of LOX Proteins

We randomly selected certain LOX proteins from each subfamily in the six species and modeled their structures using SWISS-MODEL. The 9-LOX proteins had similar structures among different species, whereas the 13-LOX proteins seemed to have different structures among these species. As shown in Figure 1, the modeled structures of the type I 13-LOX proteins in peanut and the other two species were similar. However, the structure of type II 13-LOX proteins was not conserved; there were two types, and we found that the modeled structure of type II 13-LOX in *Medicago truncatula* was different from those of the other species. The MtLOX18 lacked motifs 9 and 10, thereby resulting in the difference of modeled structure with other subfamily members (Figure 1). In general, the modeled structures of 9-LOX and type I 13-LOX subfamily proteins in different species were similar, whereas the type II 13-LOX protein had diverse structures among different species.

### Analysis of Cis-Acting Elements in Peanut LOX Genes

**Figure 4:**
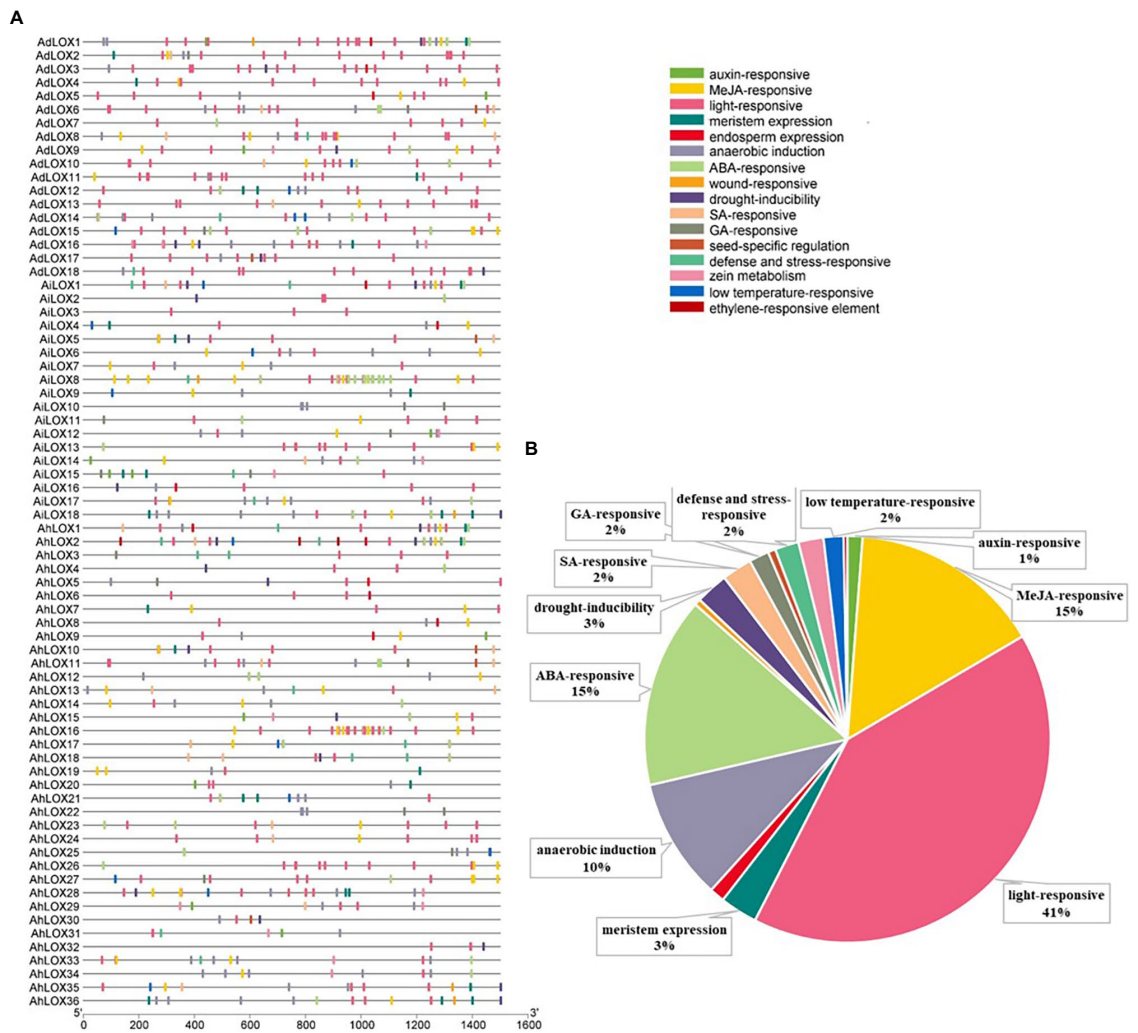
Distribution of cis-acting regulatory elements in the peanut *LOX* gene promoters. **(A)** The distribution of cis-acting elements in the promoter of each peanut *LOX*. **(B)** The ratios of different functional cis-acting elements are shown in the form of pie graphs.

Cis-acting elements in the promoter are crucial for initiating transcription and regulating gene expression. To analyze the cis-acting elements in peanut LOX gene promoters, the sequence 1.5 kb upstream from the initiation codon was predicted by searching the online PlantCARE database. The identified cis-acting elements were related to transcription, cell cycle, development, hormones as well as stresses, and most of these elements were hormone-related and stress-related elements (Figure 4). It was prediceted that the expression of peanut LOXs could be induced by hormones, such as MeJA, ABA, salicylic acid (SA), gibberellin (GA), auxin (IAA), and ethylene (ET). Moreover, many elements were involved in various stresses, such as anoxic induction, drought, zein metabolism, wounding, and low temperature. All peanut LOX genes contained many light-responsive elements. In addition, elements associated with growth and development, including meristem and endosperm expression, were found in most LOXs. Detailed information about the elements and functions in each gene were presented in Supplementary Table S4.

### Chromosomal Location and Gene Duplication of Peanut LOXs

**Figure 5:**
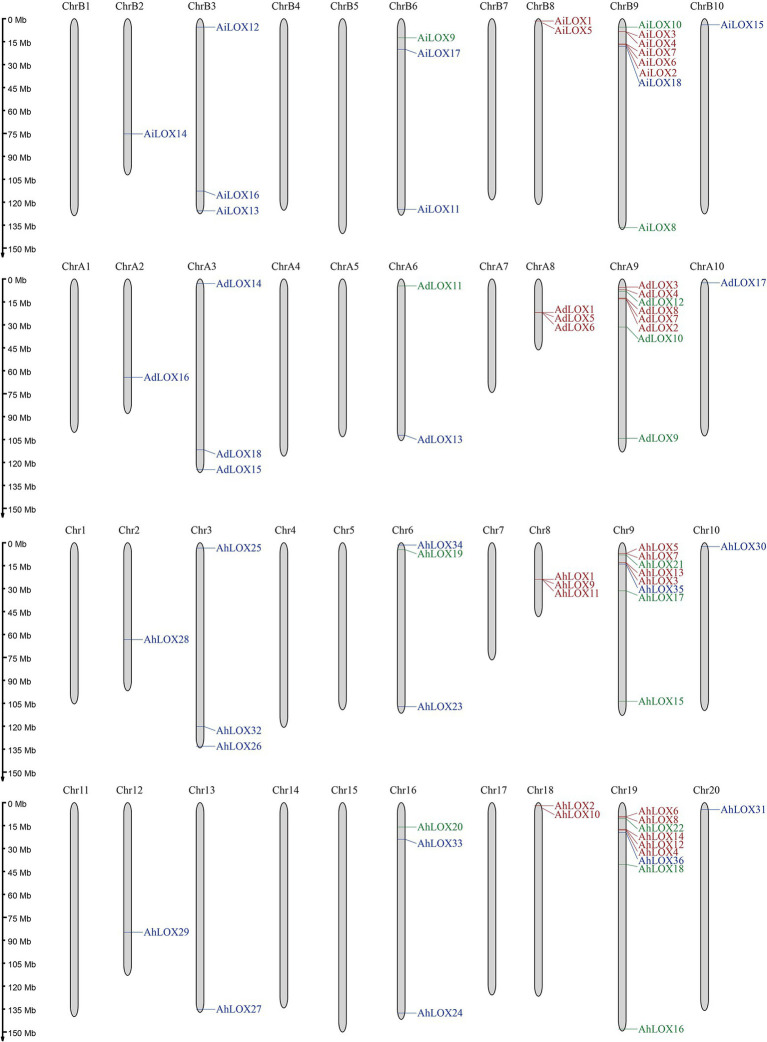
Chromosomal locations of *LOX* genes. The approximate locations of the 72 peanut *LOX* genes are displayed on the right side of the chromosomes. Genes from different subfamilies are shown in different colors (9-LOX: green, type I 13-LOX: red, and type II 13-LOX: blue).

As shown in Figure 5, the distribution of peanut LOXs on the chromosomes were not random. AdLOXs and AiLOXs were centrally distributed on chromosomes A9 and B9, respectively. Similarly, almost half of the *AhLOX* genes (17 out of 36) were located on chromosomes 9 and 19. The chromosomal locations of *AdLOX* were mapped onto 6 of the 10 *A. duranensis* (A genome) chromosomes, and *AiLOX* was also distributed on 6 of the 10 *A. ipaensis* (B genome) chromosomes. Chromosomes A3, A8, B3, B6, 3, 6, 8, and 16 each had 3 LOXs. There was only one *LOX* gene on chromosomes A2, A10, B2, B10, 2, 10, 12 13, and 20, and two *LOX* genes on chromosomes A6, B8, and 18. Chromosomes A1, A4, A5, A7, B1, B4, B5, B7, 1, 4, 5, 7, 11, 14, 15, and 17 had no LOXs. These results indicated that gene duplication events might have occurred on chromosomes A9, B9, 9, and 19 during the evolutionary process of the LOX gene family.

**Figure 6:**
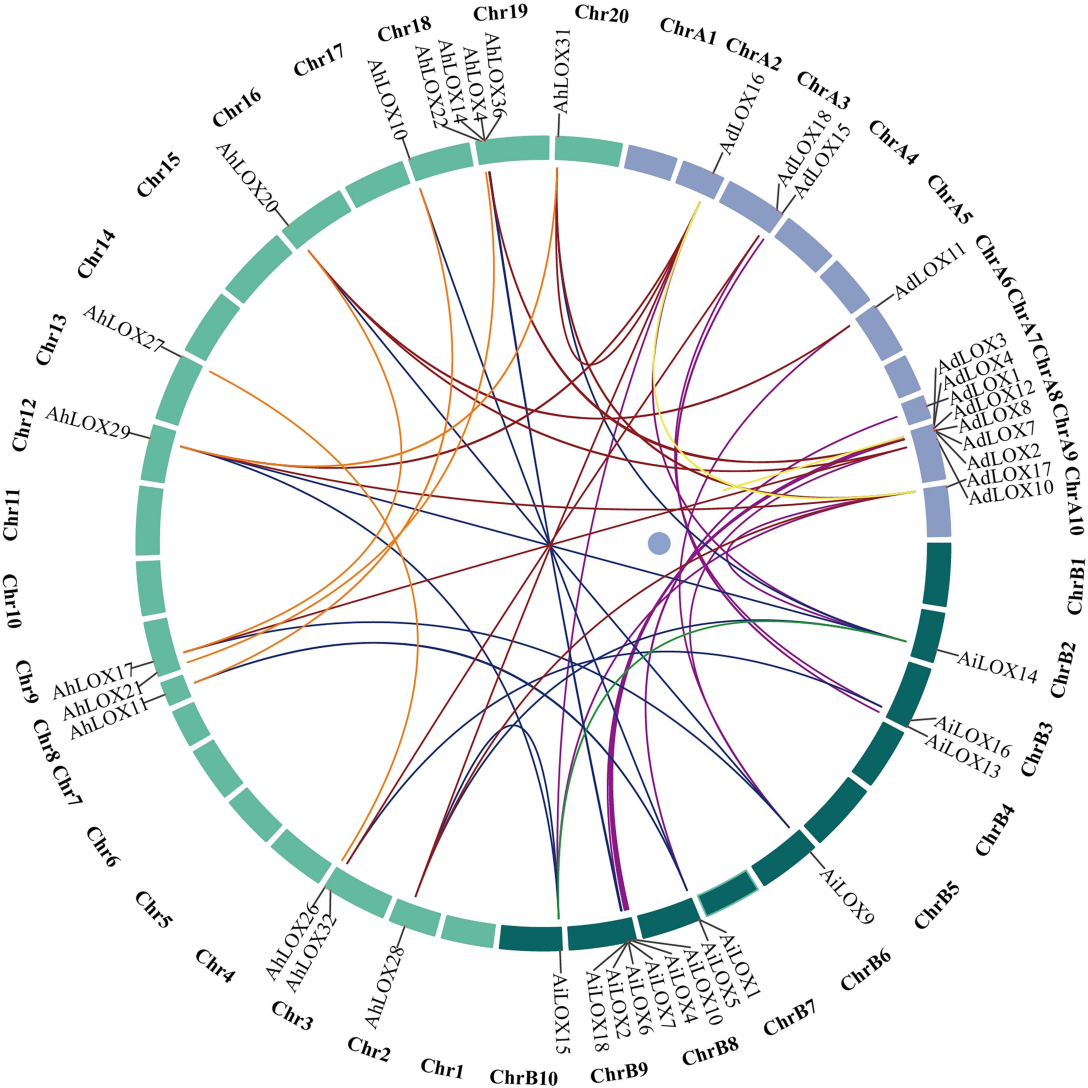
Segmentally duplicated gene pairs of LOXs in peanut. The lines highlighted in different colors indicate duplicated LOX gene pairs.

Gene duplication is widespread in the peanut genome. We analyzed the segmental duplication events of the *LOX* genes in peanut using MCScanX software. As shown in Figure 6, among 72 peanut LOXs, 48 pairs of segmental duplication genes were identified (Figure 6, Supplementary Table S5), and 14 pairs were located on chromosomes A9, B9, 9, and 19. The largest number of gene duplication events occurred in type II 13-LOX (24 pairs), followed by type I 13-LOX (14 pairs) and 9-LOX (10 pairs). These results suggested that segmental duplication played a vital role in the expansion of peanut LOX gene family.

### Tissue-Specific Expression Patterns of Peanut LOXs

**Figure 7:**
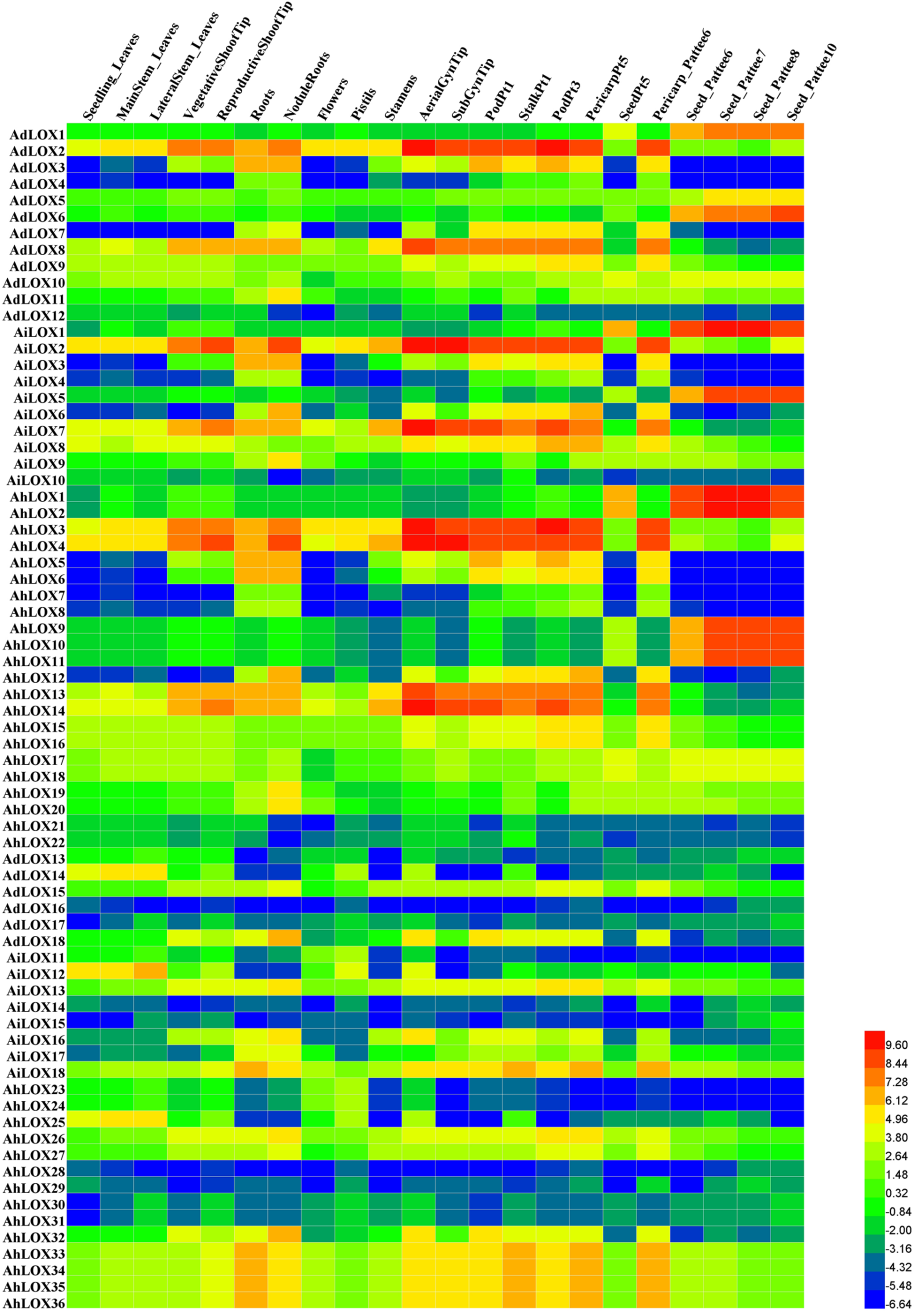
Expression profiles of *LOX* genes in 22 different peanut tissues. The heatmap was generated by Heml software, and the fragments per kilobase of transcript per million fragments (FPMK) values of peanut *LOX* genes were log2-transformed. The red and blue colors represent the maximum and minimum values, respectively.

To confirm the tissue expression patterns and potential functions of *LOX* genes in peanut growth and development, the expression levels of the genes in 22 tissues (leaves, shoot tips, roots, nodules, perianths, flowers, pistils, stamens, gynophore tips, fruits, pericarps, and seeds in nearly all developmental stages) were investigated using RNA-seq data published by Josh et al. (2016). Based on the log2-transformed FPKM values, we found that the expression levels of peanut LOXs were notably different among the 22 tissues (Figure 7). Most LOXs were expressed in all 22 tissues, except for *AdLOX4*, *AdLOX7*, *AiLOX3*, *AiLOX4*, *AiLOX6*, *AhLOX6*-*8*, *AhLOX12*, *AhLOX15*, *AhLOX23*-*25*, and *AhLOX28*. Interestingly, certain genes, *AdLOX2*, *AiLOX2*, *AiLOX8*, *AiLOX13*, *AiLOX18*, *AhLOX3*, *AhLOX4*, *AhLOX17*, *AhLOX26*, and *AhLOX33*-*36*, were expressed at extremely high levels in all the investigated tissues. Besides, a large number of genes with tissue-specific expression patterns were identified. *AdLOX1*, *AdLOX6*, *AiLOX1*, *AiLOX5*, *AhLOX1*-*2*, and *AhLOX9*-*11* were specifically highly expressed in seeds, while *AdLOX2*-*3*, *AdLOX8*, *AiLOX2*, *AiLOX7*-*8*, *AhLOX3*-*4*, and *AhLOX13*-*14* were found to be specifically expressed in the shoot tips, roots, gynophore tips, fruits, and pericarps. As shown in Figure 7 and Supplementary Table S6, most homoeologous genes, such as *AdLOX8* and *AiLOX7*, *AdLOX1* and *AiLOX1*, and *AhLOX26* and *AhLOX27*, exhibited similar expression patterns. Nevertheless, some peanut LOXs showed diverse expression patterns; for example, *AhLOX2* was specifically expressed in seeds, while its homoeologous gene *AhLOX4* was highly expressed in leaves, shoot tips, roots, nodules, perianths, flowers, pistils, stamens, gynophore tips, fruits, and pericarps. The different expression patterns of homoeologous genes may be due to the polyploidization and duplication events that occurred in peanut evolution.

### Peanut *LOX* Expression Patterns Under Abiotic Stresses

**Figure 8:**
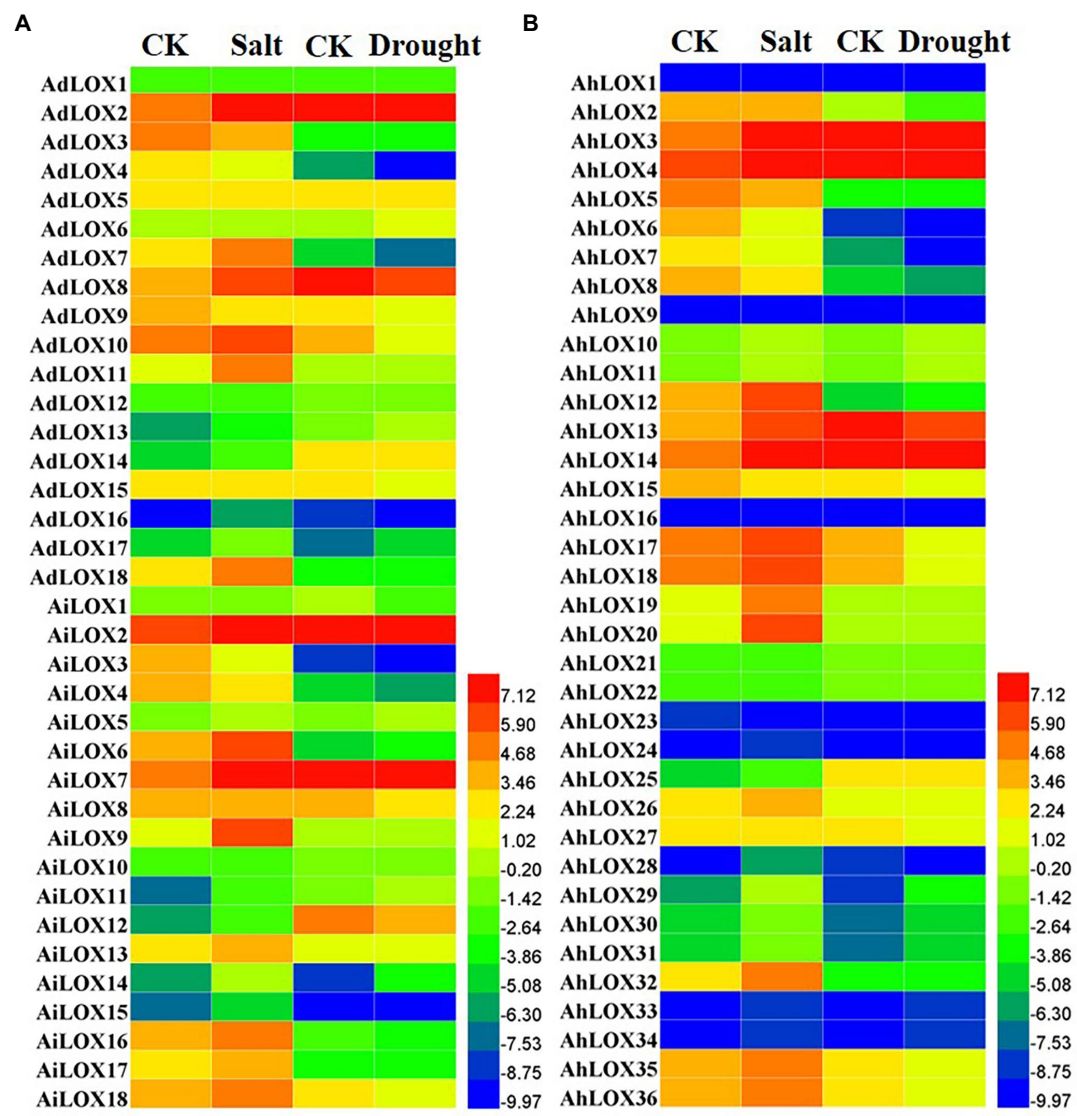
Heatmap of the expression patterns of *LOX* genes under salt and drought stress treatments in peanut **(A** and **B)**. The FPKM values of the peanut *LOX* genes were log2-transformed to create the heatmap using Heml software. The red and blue colors represent the higher or lower relative abundance of each *LOX* gene, respectively.

Previous studies have revealed that *LOX* genes play an important role in plant defenses against abiotic and biotic stresses. In this study, the expression profiles of peanut LOXs were analyzed under salt and drought stresses using published transcriptome sequencing results. The peanut *LOX* genes presented diverse expression patterns under salt and drought stress. In Figure 8, the expression of 35 peanut LOXs was upregulated more than 2-fold under salt stress, and that of 19 LOXs was upregulated more than 5-fold. Moreover, the greatest increase was up to 30-fold (*AiLOX9* and *AhLOX20*). The expression of eight peanut LOXs was downregulated nearly or more than 2-fold. Unlike the salt treatment, only a few peanut LOXs were upregulated under the drought treatment. Similarly, there were only 11 genes that were downregulated more than 2-fold after the drought treatment. It is worth noting that most of the homoeologous *LOX* genes presented similar expression patterns under salt and drought stress. In particular, the expression of *AdLOX10* decreased while the expression of *AhLOX20* stay unchanged in response to drought stress.

### Analysis of the Expression Patterns of Peanut LOXs in Response to Four Treatments by qRT-PCR

**Figure 9:**
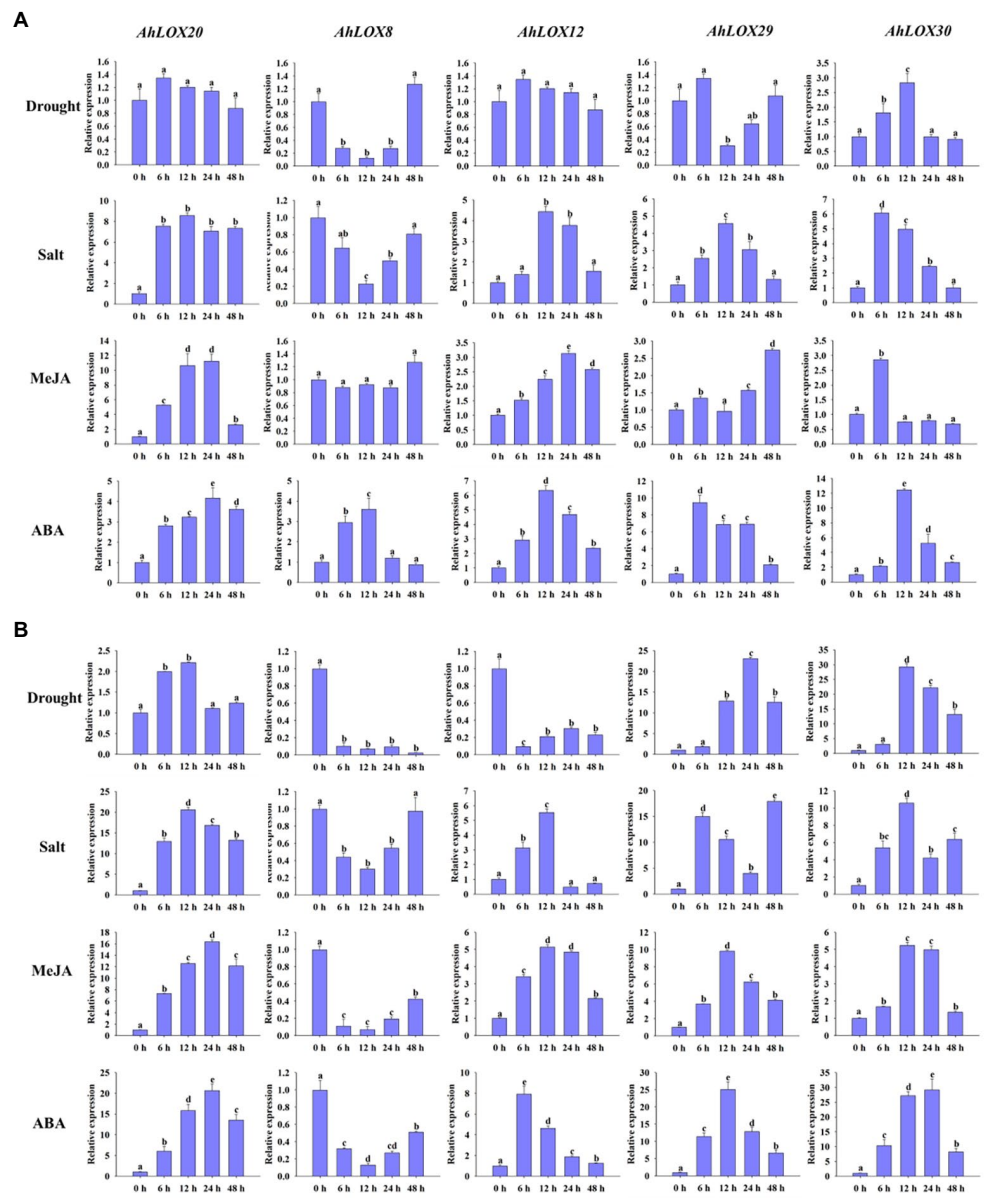
Analysis of the relative expression levels of peanut *LOX* genes by qRT-PCR. The expression profiles of five peanut *LOX* genes (*AhLOX20*, *AhLOX8*, *AhLOX12*, *AhLOX29*, and *AhLOX30*) under two stresses (drought and salt) and two hormone treatments (MeJA and ABA) were validated. **(A)** Leaves; **(B)** Roots. The data are presented as the mean ± SD (*n* = 3), and the values differed significantly at *p* < 0.05. The different letters indicate significant differences.

The qRT-PCR was used to validate the peanut *LOX* expression patterns under abiotic stresses (salt and drought) and hormone treatment (MeJA and ABA). As shown in Figure 9, five genes from different subfamilies, *AhLOX20* (9-LOX), *AhLOX8* (type I 13-LOX), *AhLOX12* (type I 13-LOX), *AhLOX29* (type II 13-LOX), and *AhLOX30* (type II 13-LOX), were investigated at six time points (0, 6, 12, 24, and 48 h) using peanut leaves (Figure 9A) and roots (Figure 9B) in the three-leaf stage.

The five selected peanut *LOX* genes exhibited different expression patterns in response to the salt, drought, MeJA and ABA treatments. After drought treatment, the expression profiles of most *LOX* genes were similar in both leaves and roots, while *AhLOX12* presented opposite expression patterns in leaves and roots. The expression of two genes (*AhLOX12* and *AhLOX30*) in leaves and that of three genes (*AhLOX20*, *AhLOX29*, and *AhLOX30*) in roots were upregulated. Remarkably, the relative expression of *AhLOX29* and *AhLOX30* was upregulated more than 20-fold in roots. A significant decrease of the relative expression of *AhLOX8* at 6 h in leaves and roots, and *AhLOX29* at 12 h in leaves after drought treatment were observed. After salt treatment, only the *AhLOX8* was downregulated in both leaves and roots. The expression levels of selected *LOX* genes (*AhLOX12*, *20*, *29*, and *30*) were increased by 4- to 8-fold in leaves. The greatest changes were found for the *AhLOX8* and *AhLOX29* in roots, which were increased up to 20-fold (Figure 9). The above-described results show that only *AhLOX12* in roots exhibited different expression patterns in response to drought and salt stress. In addition, the relative expression level of *AhLOX29* in roots was increased by approximately 20-fold in response to both drought and salt treatments. As for the MeJA and ABA treatments, the expression of all the genes except *AhLOX8* was upregulated, and the maximum change was nearly 30-fold (*AhLOX30* after ABA treatment) in roots. Both MeJA and ABA stress led to the downregulation of *AhLOX8* in roots, and ABA treatment resulted in upregulation of *AhLOX8* in leaves. Together, the relative expression of three genes, *AhLOX20* (9-LOX), *AhLOX29* (type II 13-LOX), and *AhLOX30* (type II 13-LOX), increased in response to the four treatments (drought, salt, MeJA, and ABA). The expression of other *AhLOX* gene members, such as *AhLOX8* (type I 13-LOX), was decreased after exposure to these four stresses. The expression of *AhLOX12* (type II 13-LOX) was upregulated by salt, MeJA, and ABA treatment and downregulated by exposure to drought.

### Overexpression of the *AhLOX29* Gene in *Arabidopsis* Enhances Resistance to Drought

**Figure 10:**
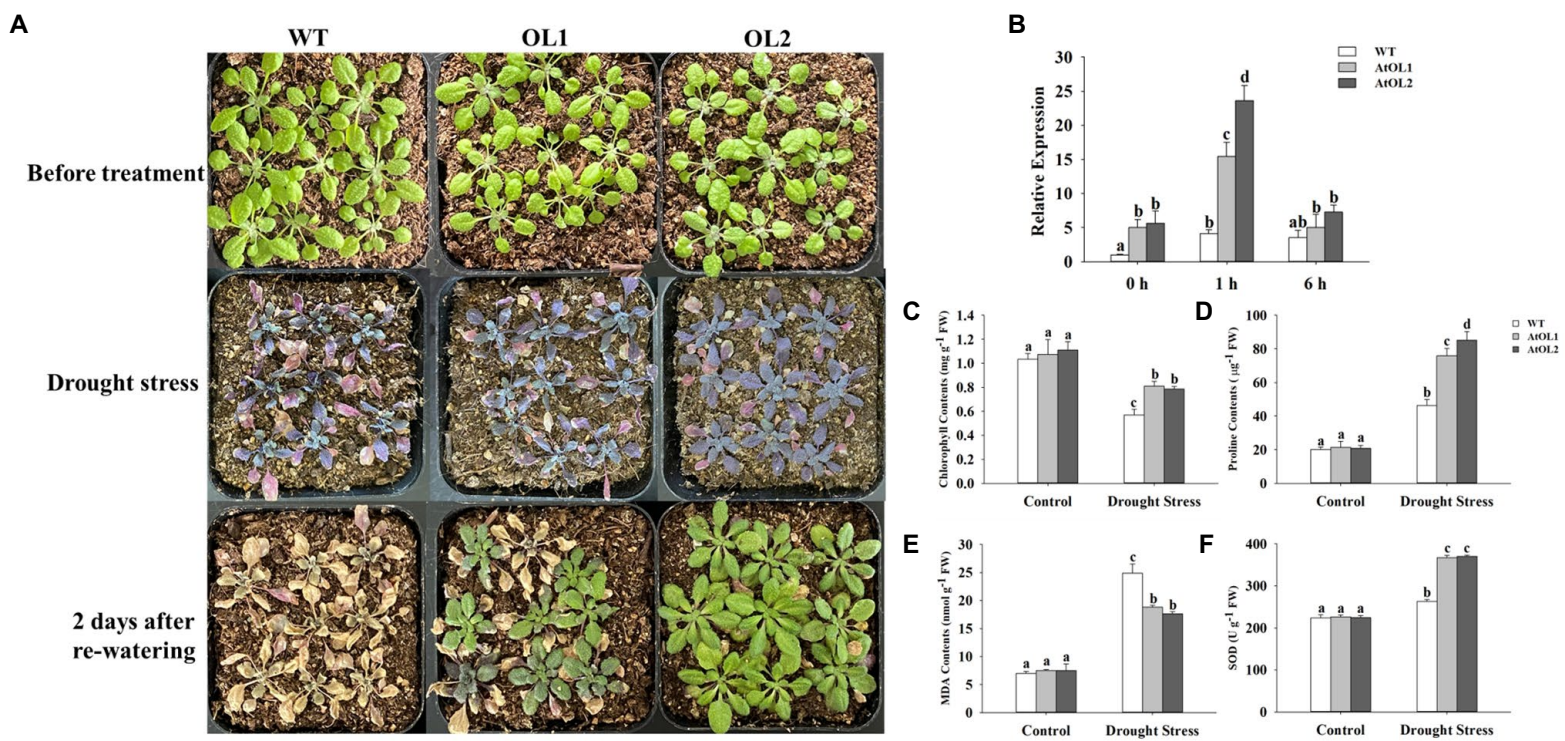
Overexpression of *AhLOX29* improved tolerance to drought stress in transgenic Arabidopsis. **(A)** Phenotype of control and transgenic plants before and after drought stress treatment; **(B)** the relative expression level of *AhLOX29* in WT and transgenic plants; **(C)** chlorophyll contents; **(D)** proline contents; **(E)** MDA contents and **(F)** SOD activity.

WT and transgenic *Arabidopsis* were exposed to drought stress during the seedling stage, and the drought tolerance of the *AhLOX29* transgenic lines was significantly improved (Figure 10A). The relative expression levels of *AhLOX29* in the transgenic and WT lines were measured by qRT-PCR. Results showed that the relative expression levels of the two overexpression lines were significantly higher than that of the WT lines (Figure 10B), indicating that the *AhLOX29* was successfully transcribed in *Arabidopsis*. After being treated with 20% PEG6000 for 1 h, the expression of *AhLOX29* in the AhOL and WT lines was obviously increased, and the transcript levels of *AhLOX29* in the AtOL lines were significantly higher than those in the WT (Figure 10B). Compared with 1 h, the expression of the *AhLOX29* in the WT was unchanged after 6 h of 20% PEG6000 treatment, but the expression of AtOL1 and AtOL2 was decreased at 6 h (Figure 10B). To briefly sum up, the *AhLOX29* is a stress-responsive gene, and the rapid accumulation of *AhLOX29* at 1 h suggests its positive role in drought resistance.

The total chlorophyll, proline and MDA contents as well as SOD activity in the leaves on day 0 and day 10 after drought stress were measured (Figures 10C–F). After 10 days of drought treatment, the chlorophyll content in leaves decreased, and the chlorophyll contents in the gene-overexpressed plants were significantly higher than those in the WT plants (Figure 10C). The proline content in plants increased under drought challenges, thereby functioning as an osmotic protective agent to alleviate or resist the damage caused by drought stress. After drought stress, the proline content in *Arabidopsis* leaves increased by approximately 2-fold in the WT plants and 4-fold in the transgenic plants, and the gene-overexpressed lines accumulated higher levels of proline than the WT (Figure 10D). The MDA contents were remarkably increased by drought stress, and higher MDA resulted in greater damage to the membrane and plants. The MDA contents in the WT and transgenic *Arabidopsis* lines were equal before drought treatment, and drought treatment significantly increased the MDA contents from 6.99 to 24.85 nmol/g in the WT plants and to 18.8 and 17.6 nmol/g in the AtOL1 and AtOL2 lines, respectively (Figure 10E). Under normal conditions, no difference in SOD activity were found between the WT and transgenic plants (Figure 10F). However, after drought challenge for 10 days, the SOD activity was obviously increased, and gene-overexpressed *Arabidopsis* displayed a notable increase in SOD activity compared with WT *Arabidopsis*.

## Discussion

To date, the LOX multigene family has been identified and functionally characterized in various plant species, such as *Arabidopsis* (Umate, 2011), rice (Umate, 2011), cotton (Shaban et al., 2018), tomato (Upadhyay et al., 2019), and pepper (Sarde et al., 2018). Furthermore, previous studies in these species have focused mainly on its roles in protein structure, evolution, growth and development, improvement of seed quality as well as defense responses to biotic and abiotic stresses (Feussner and Wasternack, 2002; Porta and Rocha-Sosa, 2002; Roy Chowdhury et al., 2016). Although several genes related to the molecular characterization of peanut LOXs have been reported, a genome-wide identification of the LOX gene family in cultivated peanut and the functional analysis of these genes under abiotic stress conditions have not been performed. The fully annotated reference genomes of *A. duranensis* and *A. ipaensis* have been released (Bertioli et al., 2016), and whole-genome sequences of cultivated peanut (*Tifrunner*) were recently available as well (Bertioli et al., 2019). Accordingly, it becomes possible to identify all the putative *LOX* genes in peanut and analyze potential functions.

Unlike the other plant species investigated in this study, the number of AhLOXs in peanut (36) was significantly higher than that of AtLOXs (6), AiLOXs (18), and AdLOXs (18). The high numbers of AhLOXs might be attributed to the allotetraploid genome of peanut, which experienced whole-genome duplication and gene duplication events (Bertioli et al., 2011). Gene duplication and whole genome duplication events contributed to the expansion of gene families in plant evolution (Cannon et al., 2004). In addition, a collinearity analysis showed that there were three groups of tandem duplications, and 48 pairs of segmental duplication genes were identified in peanut. Our results regarding gene duplication events further elucidated the mechanism underlying the expansion of the LOX gene family. New genes might have been generated during such duplication events, thereby resulting in the generation of new biological functions (Conant and Wolfe, 2008).

Based on the protein structure, sequence similarity and presence of a chloroplast transit peptide (Feussner and Wasternack, 2002), peanut LOXs were classified into three subfamilies *via* phylogenetic analysis, 9-LOX, type I 13-LOX, and type II 13-LOX. The gene structure analysis indicated that most of these peanut genes had similar structures. Intron gain or loss was observed in several genes in the 9-LOX, type I 13-LOX, and type II 13-LOX subfamilies, which might be due to the selection pressures during peanut evolution (Zhang, 2003; Babenko et al., 2004). Accordingly, the diverse exon–intron structure contributed to their functional diversification (Mattick, 1994). Moreover, we gained additional insights into protein motifs in peanut (Figure 3). As expected, the motifs of the peanut LOXs were conserved, demonstrating that these conserved motifs might play key roles in the conservation of *LOX* gene functions. The predicted 3D structures of proteins were also determined to compare protein structures within the three subfamilies in different species (Figure 1). The modeled structures of four genes (*AhLOX1*, *AhLOX28*, *AdLOX13*, and *AiLOX17*), which lacked motifs 1, 4, 5, and 10, were looser than those possessing all of the 10 motifs (Supplementary Table S3). In addition, the structures of these four genes were different from each other as well, and the variation was likely to be the main factor leading to a variety of protein functions. Taken together, the structure and phylogeny of the LOX gene family showed diversity and complexity, thereby contributing to functional diversification.

The results for cis-acting elements revealed that peanut LOXs might participate in regulating various biological and molecular processes, such as transcription, cell cycle, development, hormones, and biotic/abiotic stresses. A large number of cis-acting regulatory elements related to hormones (MeJA, ABA, SA, GA, IAA, ET, etc.) and various stresses (anoxic conditions, drought, zein metabolism, wounding, low temperature, etc.) were identified in each peanut *LOX* promoter. Our findings were in consistent with previous studies which proved that *LOX* played multiple roles in cotton (Shaban et al., 2018) and tomato (Upadhyay et al., 2019). A recent study demonstrated that the cis-acting elements of LOX genes could regulate the response to abiotic stresses in tomato (Upadhyay et al., 2019). In addition, two previous studies revealed that the *LOX* gene played an important role in resistance to root-knot nematodes (Gao et al., 2008) and phloem feeders (Zhou et al., 2009). Therefore, this analysis further proved that the LOXs belonged to a multifunctional gene family, and they were involved in defense responses to various environmental stresses as well as in plant growth and development.

The expression patterns of peanut *LOX* genes were analyzed using public RNA-seq data for 22 tissues. The 72 candidate *LOX* genes exhibited prominently different expression profiles in 22 tissues, and even genes in the same subfamily were expressed differently. Thus it could be summarized that the *LOX* genes performed diverse and crucial functions in different growth and developmental stages. For instance, LOXs played a dual role in the fruit development of tomato; the three tomato *LOX* genes were separately regulated during fruit ripening, and the expressions could be influenced by ethylene and developmental factors (Griffiths et al., 1999). In *Arabidopsis*, *LOX* genes have been evidenced to participate in the developmental transition from vegetative to flowering, lateral root development and stress-induced senescence (Feng et al., 2012). Hence, different LOXs could regulate various physiological processes in different tissues by specific expression patterns.

Lipoxygenases were a multifunctional gene family, which was confirmed *via* the RNA-seq data and qRT-PCR results in this study. We found that peanut LOXs exhibited different expression patterns in response to abiotic stresses and hormone treatments, suggesting that they might be involved in various plant stress response pathways and closely related to hormonal regulation. As for rice, *OsLOX*-silenced plants were more susceptible to striped stem borers, which were chewing herbivores, but more resistant to brown planthoppers, which were phloem feeders; thus, the *LOX* played opposite roles with regard to resistance to chewing and phloem-feeding herbivores (Zhou et al., 2009). Additionally, the *LOX* participated in herbivore-induced JA biosynthesis, and the modulation of JA activity was essential for the plant defense responses (Zhou et al., 2009). It was also corroborated that the rice *LOX3* gene could function in response to drought and pathogens (Liu et al., 2008). In peanut, a number of *LOX* genes were functionally characterized according to the response to *Aspergillus*-seed interactions. For instance, *PnLOX1* and *PnLOX2*/*PnLOX3* were either positively or negatively regulated during *Aspergillus* infection, and these genes were proved to be one of the host genetic factors influencing the aflatoxin contamination (Korani et al., 2018). In mature peanut seeds, *AhLOX1* (*PnLOX1*) expression could be highly induced by *Aspergillus* spp., or by MeJA and wounding (Burow et al., 2000). Although multifunctional LOX gene families have been found in many plants, the functional characterization of LOXs in peanut was rarely investigated.

Drought is one of the key stresses that has negative impact on crop yield (Dietz et al., 2021). Above results from the analysis of cis-acting elements in promoters, gene expression profiles, and transgenic plants suggested the potential role of *AhLOX* genes in drought resistance. Overexpression of *AhLOX29* significantly enhanced the resistance to drought in *Arabidopsis* (Figure 10). Supporting our findings, previous studides revealed that *LOX6* was essential for the production of 12-oxo-phytodienoic acid in leaves and roots in response to osmotic stress and drought (Grebner et al., 2013; Savchenko et al., 2014). Furthermore, jasmonates produced in roots were independent in leaves, which was demonstrated by grafting experiments with jasmonate-deficient mutants (Grebner et al., 2013). Osmotic and salt stresses could trigger the overproduction of reactive oxygen species (ROS) in plants, and the scavenging of ROS was an important mechanism for the resistance to abiotic stresses (Ullah et al., 2017). MDA is widely used as an indicator of drought exposure to evaluate the degree of plasma membrane damage and the drought tolerance capacity of plants (Zhang et al., 2021). The decrease of chlorophyll contents in transgenic *Arabidopsis* was obviously lower than that in WT, which indicated that the overexpression of the *AhLOX29* gene could reduce the negative effect of drought stress on *Arabidopsis* photosynthesis. Compared with the WT, the OE-*AhLOX29* lines had higher SOD activities and lower MDA contents, suggesting the improved capacity to scavenge ROS, thereby protected the membrane from being damaged in transgenic plants. Hence, the *AhLOX29* gene could enhance the resistance to drought stress in *Arabidopsis*. Peanut *LOX* genes are of critical significance for peanut growth and development as well as the response to various stresses.

## Conclusion

In this study, 72 *LOX* genes in the whole peanut genome were identified and classified into 9-LOX, type I 13-LOX and type II 13-LOX subfamilies. The investigation of phylogenetic relationships, gene structures and protein motifs revealed that the structure and function of most LOXs in peanut were relatively conserved during evolution. The *LOX* genes are nonrandomly located on chromosomes, and most of them are located on chromosomes A9, B9, 9, and 19. The segmentally duplicated genes contribute significantly to the expansion of the LOX gene family. Peanut *LOX* genes are involved in various biological processes, including growth and development, hormones (MeJA, ABA, SA, and JA) and responses to multiple stresses (drought, salt, anoxic induction, wounding, and low temperature). Overexpression of *AhLOX29* in *Arabidopsis* increased the tolerance to drought stress possibly by scavenging ROS and alleviating membrane damage. The systematic and comprehensive data from this study not only provide a foundation for revealing the functional roles of peanut *LOX* genes but also contribute to the selection of appropriate candidate *LOX* genes for further characterization with regard to plant development and stress responses.

## Data Availability Statement

The datasets presented in this study can be found in online repositories. The names of the repository/repositories and accession number(s) can be found in the article/Supplementary Material.

## Author Contributions

YM and SS conceived and designed the study. YM performed the experiments and wrote the manuscript. QS helped the bioinformatics analysis. CY provided the plant materials. JW, XZ, CY, and CL revised the paper. All authors have read and approved the final version of the manuscript.

## Funding

This research was supported by the innovation Project of SAAS (CXGC2021B33) and (CXGC2021A30), the National Natural Science Foundation of China (31901506), the Taishan Scholar Project of Shandong Province (ts201712080), the Agro-industry Technology Research System of Shandong Province (SDAIT-04\u201302), the Natural Science Foundation of Shandong Province (ZR2021MC128) and Shandong Elite Variety Project (2020LZGC001). The funders had no role in the design and data analysis of this study, and in writing the manuscript.

## Conflict of Interest

The authors declare that the research was conducted in the absence of any commercial or financial relationships that could be construed as a potential conflict of interest.

## Publisher’s Note

All claims expressed in this article are solely those of the authors and do not necessarily represent those of their affiliated organizations, or those of the publisher, the editors and the reviewers. Any product that may be evaluated in this article, or claim that may be made by its manufacturer, is not guaranteed or endorsed by the publisher.

## Footnotes

^1^ https://www.peanutbase.org/

^2^ http://pfam.xfam.org/

^3^ http://pfam.xfam.org/

^4^ http://www.ebi.ac.uk/interpro/scan.html

^5^ https://www.ncbi.nlm.nih.gov/Structure/bwrpsb/bwrpsb.cgi

^6^ https://web.expasy.org/protparam/

^7^ http://molevol.cmima.csic.es/castresana/Gblocks_server.html

^8^ http://www.evolgenius.info/evolview/

^9^ http://gsds.cbi.pku.edu.cn/

^10^ http://meme-suite.org/

^11^ http://bioinformatics.psb.ugent.be/webtools/plantcare/html/

^12^ https://beta.swissmodel.expasy.org/

^13^ https://www.peanutbase.org/

^14^ http://chibba.pgml.uga.edu/mcscan2/

^15^ http://circos.ca/

^16^ https://www.peanutbase.org/

## References

[ref1] Andrew W. Martino B. Stefan B. Gabriel S. Gerardo T. Rafal G.. (2018). SWISS-MODEL: homology modelling of protein structures and complexes. Nucleic Acids Res. 46, W296–W303. doi: 10.1093/nar/gky427, PMID: 29788355 PMC6030848

[ref2] Babenko V. N. Rogozin I. B. Mekhedov S. L. Koonin E. V. (2004). Prevalence of intron gain over intron loss in the evolution of paralogous gene families. Nucleic Acids Res. 32, 3724–3733. doi: 10.1093/nar/gkh686, PMID: 15254274 PMC484173

[ref3] Bannenberg G. Martinez M. Hamberg M. Castresana C. (2009). Diversity of the enzymatic activity in the Lipoxygenase gene family of *Arabidopsis thaliana*. Lipids 44, 85–95. doi: 10.1007/s11745-008-3245-7, PMID: 18949503

[ref4] Bates L. S. Waldren R. P. Teare I. D. (1973). Rapid determination of free proline for water-stress studies. Plant Soil 39, 205–207. doi: 10.1007/BF00018060, PMID: 20688380

[ref5] Bell E. Creelman R. A. Mullet J. E. (1995). A chloroplast lipoxygenase is required for wound-induced jasmonic acid accumulation in *Arabidopsis*. Proc. Natl. Acad. Sci. U. S. A. 92, 8675–8679. doi: 10.1073/pnas.92.19.8675, PMID: 7567995 PMC41029

[ref6] Bertioli D. J. Cannon S. B. Froenicke L. Huang G. Farmer A. D. Cannon E. K.. (2016). The genome sequences of *Arachis duranensis* and *Arachis ipaensis*, the diploid ancestors of cultivated peanut. Nat. Genet. 48, 438–446. doi: 10.1038/ng.3517, PMID: 26901068

[ref7] Bertioli D. J. Jenkins J. Clevenger J. Dudchenko O. Schmutz J. (2019). The genome sequence of segmental allotetraploid peanut *Arachis hypogaea*. Nat. Genet. 51, 877–884. doi: 10.1038/s41588-019-0405-z, PMID: 31043755

[ref8] Bertioli D. J. Seijo G. Freitas F. O. Valls J. Leal-Bertioli S. Moretzsohn M. C. (2011). An overview of peanut and its wild relatives. Plant Genet. Res. 9, 134–149. doi: 10.1017/S1479262110000444

[ref9] Brash A. R. (1999). Lipoxygenases: occurrence, functions, catalysis, and acquisition of substrate. J. Biol. Chem. 274, 23679–23682. doi: 10.1074/jbc.274.34.23679, PMID: 10446122

[ref10] Burow G. B. Gardner H. W. Keller N. P. (2000). A peanut seed lipoxygenase responsive to *Aspergillus* colonization. Plant Mol. Biol. 42, 689–701. doi: 10.1023/A:1006361305703, PMID: 10809442

[ref11] Caldelari D. Wang G. Farmer E. E. Dong X. (2011). Arabidopsis *lox3 lox4* double mutants are male sterile and defective in global proliferative arrest. Plant Mol. Biol. 75, 25–33. doi: 10.1007/s11103-010-9701-9, PMID: 21052784

[ref12] Cannon S. B. Mitra A. Baumgarten A. Young N. D. May G. (2004). The roles of segmental and tandem gene duplication in the evolution of large gene families in *Arabidopsis thaliana*. BMC Plant Biol. 4, 10. doi: 10.1186/1471-2229-4-10, PMID: 15171794 PMC446195

[ref13] Carrera A. Echenique V. Zhang W. Helguera M. Manthey F. Schrager A.. (2007). A deletion at the Lpx-B1 locus is associated with low lipoxygenase activity and improved pasta color in durum wheat (*Triticum turgidum ssp. durum*). J. Cereal Sci. 45, 67–77. doi: 10.1016/j.jcs.2006.07.001

[ref14] Christensen S. A. Huffaker A. Kaplan F. Sims J. Ziemann S. Doehlemann G.. (2015). Maize death acids, 9-lipoxygenase-derived cyclopente(a)nones, display activity as cytotoxic phytoalexins and transcriptional mediators. Proc. Natl. Acad. Sci. U. S. A. 112, 11407–11412. doi: 10.1073/pnas.1511131112, PMID: 26305953 PMC4568653

[ref15] Christensen S. A. Nemchenko A. Borrego E. Murray I. Sobhy I. S. Bosak L.. (2013). The maize lipoxygenase, *ZmLOX10*, mediates green leaf volatile, jasmonate and herbivore-induced plant volatile production for defense against insect attack. Plant J. 74, 59–73. doi: 10.1111/tpj.12101, PMID: 23279660

[ref16] Conant G. C. Wolfe K. H. (2008). Turning a hobby into a job: how duplicated genes find new functions. Nat. Rev. Genet. 9, 938–950. doi: 10.1038/nrg2482, PMID: 19015656

[ref17] Dietz K.-J. Zörb C. Geilfus C.-M. (2021). Drought and crop yield. Plant Biol. 23, 881–893. doi: 10.1111/plb.13304 34396653

[ref18] Feng B. Dong Z. Xu Z. Wang D. Wang T. (2012). Molecular characterization of a novel type of lipoxygenase (LOX) gene from common wheat (*Triticum aestivum L*.). Mol. Breed. 30, 113–124. doi: 10.1007/s11032-011-9603-9

[ref19] Feussner I. Wasternack C. (2002). The lipoxygenase pathway. Annu. Rev. Plant Biol. 53, 275–297. doi: 10.1146/annurev.arplant.53.100301.135248, PMID: 12221977

[ref20] Finn R. D. Jaina M. Benjamin S. B. C. Sam G. J. Volker H. Timo L.. (2006). Pfam: clans, web tools and services. Nucleic Acids Res. 34, D247–D251. doi: 10.1093/nar/gkj149, PMID: 16381856 PMC1347511

[ref21] Gao X. Starr J. Goebel C. Engelberth J. Feussner I. Tumlinson J.. (2008). Maize 9-lipoxygenase ZmLOX3 controls development, root-specific expression of defense genes, and resistance to root-knot nematodes. Mol. Plant-Microbe Interact. 21, 98–109. doi: 10.1094/MPMI-21-1-0098, PMID: 18052887

[ref22] Gasteiger E. Hoogland C. Gattiker A. Duvaud S. E. Wilkins M. R. Appel R. D.. (1999). Protein identification and analysis tools on the ExPASy server. Methods Mol. Biol. 112:531. doi: 10.1385/1.59259-584-7:531 10027275

[ref23] Grebner W. Stingl N. E. Oenel A. Mueller M. J. Berger S. (2013). Lipoxygenase6-dependent Oxylipin synthesis in roots is required for abiotic and biotic stress resistance of *Arabidopsis*. Plant Physiol. 161, 2159–2170. doi: 10.1104/pp.113.214544, PMID: 23444343 PMC3613484

[ref24] Griffiths A. Barry C. Alpuche-Solis A. G. Grierson D. (1999). Ethylene and developmental signals regulate expression of lipoxygenase genes during tomato fruit ripening. Front. Plant Sci. 50:793. doi: 10.1093/jexbot/50.335.793

[ref25] Guo A.-Y. Zhu Q.-H. Chen X. Luo J.-C. (2007). GSDS: a gene structure display server. Yichuan 29, 1023–1026. PMID: 17681935

[ref26] Hao L. Wang Y. Zhang J. Xie Y. Zhang M. Duan L.. (2013). Coronatine enhances drought tolerance via improving antioxidative capacity to maintaining higher photosynthetic performance in soybean. Plant Sci. 210, 1–9. doi: 10.1016/j.plantsci.2013.05.006, PMID: 23849108

[ref27] Heitz T. Bergey D. R. Ryan C. A. (1997). A gene encoding a chloroplast-targeted lipoxygenase in tomato leaves is transiently induced by wounding, systemin, and methyl jasmonate. Plant Physiol. 114, 1085–1093. doi: 10.1104/pp.114.3.1085, PMID: 9232884 PMC158398

[ref28] Hui D. Lai J. Qian W. Zhang S. Liang C. Dai Y. S.. (2016a). Jasmonate complements the function of *Arabidopsis* lipoxygenase3 in salinity stress response. Plant Sci. 244, 1–7. doi: 10.1016/j.plantsci.2015.11.009, PMID: 26810448

[ref29] Hui S. Wang P. Li C. Han S. Wang X. (2016b). Identification of lipoxygenase (LOX) genes from legumes and their responses in wild type and cultivated peanut upon *Aspergillus flavus* infection. Sci. Rep. 6:35245. doi: 10.1038/srep35245, PMID: 27731413 PMC5059700

[ref30] Jin H. S. Van K. Dong H. K. Kim K. D. Jang Y. E. Choi B. S.. (2008). The lipoxygenase gene family: a genomic fossil of shared polyploidy between *Glycine max* and *Medicago truncatula*. BMC Plant Biol. 8:133. doi: 10.1186/1471-2229-8-133, PMID: 19105811 PMC2644698

[ref31] Josh C. Chu Y. Brian S. Peggy O. A. (2016). A developmental Transcriptome map for Allotetraploid *Arachis hypogaea*. Front. Plant Sci. 7:1446. doi: 10.3389/fpls.2016.01446, PMID: 27746793 PMC5043296

[ref32] Khan S. A. Zhang C. Ali N. Gandeka M. (2020). Highdensity SNP map facilitates fine mapping of QTLs and candidate genes discovery for Aspergillus favus resistance in peanut (Arachis hypogaea). Theor. Appl. Genet. 133, 2239–2257. doi: 10.1007/s00122-020-03594-0 32285164

[ref33] Kolomiets M. V. Hannapel D. J. Chen H. Tymeson M. Gladon R. J. (2001). Lipoxygenase is involved in the control of potato tuber development. Plant Cell 13, 613–626. doi: 10.1105/tpc.13.3.613, PMID: 11251100 PMC135504

[ref34] Korani W. Chu Y. Holbrook C. C. Ozias-Akins P. (2018). Insight into genes regulating post-harvest Aflatoxin contamination of Tetraploid Peanut from transcriptional profiling. Genetics 209, 143–156. doi: 10.1534/genetics.118.300478, PMID: 29545468 PMC5937179

[ref35] Liu M. Chervin C. (1997). Ethylene and fruit ripening. Physiol. Plant. 101, 727–739. doi: 10.1111/j.1399-3054.1997.tb01057.x

[ref36] Liu N. Jiang L. Zhang W. Liu L. Zhai H. Wan J. (2008). Role of LOX3 gene in alleviating adverse effects of drought and pathogens in Rice. Rice Sci. 15, 276–282. doi: 10.1016/S1672-6308(09)60004-4

[ref37] Liu L. Sonbol F.-M. Huot B. Gu Y. Withers J. Mwimba M.. (2016). Salicylic acid receptors activate jasmonic acid signalling through a non-canonical pathway to promote effector-triggered immunity. Nat. Commun. 7. doi: 10.1038/ncomms13099, PMID: 27725643 PMC5062614

[ref38] Marchler-Bauer A. Anderson J. Cherukuri P. Dewweese-Scott C. Geer L. Gwadz M.. (2005). CDD: a conserved domain database for protein classification. Nucleic Acids Res. 33, D192–D196. doi: 10.1093/nar/gki069, PMID: 15608175 PMC540023

[ref39] Mariutto M. (2011). The elicitation of a systemic resistance by pseudomonas putida BTP1 in tomato involves the stimulation of two lipoxygenase isoforms. BMC Plant Biol. 11, 29–29. doi: 10.1186/1471-2229-11-29, PMID: 21294872 PMC3042376

[ref40] Mattick J. S. (1994). Introns: evolution and function. Curr. Opin. Genet. Dev. 4, 823–831. doi: 10.1016/0959-437X(94)90066-3, PMID: 7888751

[ref41] Milligan S. J. Yaghoobi J. Kaloshian I. Zabel P. Williamson V. M. (1998). The root knot nematode resistance gene mi from tomato is a member of the leucine zipper, nucleotide binding, leucine-rich repeat family of plant genes. Plant Cell 10, 1307–1319. doi: 10.1105/tpc.10.8.1307, PMID: 9707531 PMC144378

[ref42] Müller V. Amé M. Carrari F. Gieco J. O. Asis R. (2014). Lipoxygenase activation in Peanut seed cultivars resistant and susceptible to *Aspergillus* parasiticus colonization. Phytopathology 104, 1340–1348. doi: 10.1094/PHYTO-12-13-0338-R, PMID: 24941329

[ref43] Ogunola O. F. Hawkins L. K. Mylroie E. Kolomiets M. V. Borrego E. Tang J. D.. (2017). Characterization of the maize lipoxygenase gene family in relation to aflatoxin accumulation resistance. PLoS One 12, e0181265. doi: 10.1371/journal.pone.0181265, PMID: 28715485 PMC5513560

[ref44] Ozalvo R. Cabrera J. Escobar C. Christensen S. A. Borrego E. J. Kolomiets M. V.. (2014). Two closely related members of Arabidopsis 13-lipoxygenases (13-LOXs), *LOX3* and *LOX4*, reveal distinct functions in response to plant-parasitic nematode infection. Mol. Plant Pathol. 15, 319–332. doi: 10.1111/mpp.12094, PMID: 24286169 PMC6638665

[ref45] Porta H. Rocha-Sosa M. (2002). Plant lipoxygenases. Physiological and molecular features. Plant Physiol. 130, 15–21. doi: 10.1104/pp.010787, PMID: 12226483 PMC1540254

[ref46] Quevillon E. Silventoinen V. Pillai S. Harte N. Mulder N. Apweiler R.. (2005). InterProScan: protein domains identifier. Nucleic Acids Res. 33, W116–W120. doi: 10.1093/nar/gki442, PMID: 15980438 PMC1160203

[ref47] Rayko H. Baldwin I. T. (2010). Antisense LOX expression increases herbivore performance by decreasing defense responses and inhibiting growth-related transcriptional reorganization in *Nicotiana attenuata*. Plant J. Cell Mol. Biol. 36, 794–807. doi: 10.1046/j.1365-313X.2003.01921.x, PMID: 14675445

[ref48] Reymond P. Weber H. Damond M. Farmer E. E. (2000). Differential gene expression in response to mechanical wounding and insect feeding in *Arabidopsis*. Plant Cell 12, 707–719. doi: 10.1105/tpc.12.5.707, PMID: 10810145 PMC139922

[ref49] Roy Chowdhury M. Li X. Qi H. Li W. Sun J. Huang C.. (2016). Functional characterization of 9−/13-LOXs in Rice and silencing their expressions to improve grain qualities. Biomed. Res. Int. 2016, 1–8. doi: 10.1155/2016/4275904, PMID: 27403427 PMC4925972

[ref50] Sarde S. J. Kumar A. Remme R. N. Dicke M. (2018). Genome-wide identification, classification and expression of lipoxygenase gene family in pepper. Plant Mol. Biol. 98, 375–387. doi: 10.1007/s11103-018-0785-y, PMID: 30317456 PMC6244800

[ref51] Savchenko T. Kolla V. A. Wang C.-Q. Nasafi Z. Hicks D. R. Phadungchob B.. (2014). Functional convergence of Oxylipin and Abscisic acid pathways controls Stomatal closure in response to drought. Plant Physiol. 164, 1151–1160. doi: 10.1104/pp.113.234310, PMID: 24429214 PMC3938610

[ref52] Shaban M. Ahmed M. M. Sun H. Ullah A. Zhu L. (2018). Genome-wide identification of lipoxygenase gene family in cotton and functional characterization in response to abiotic stresses. BMC Genomics 19, 599. doi: 10.1186/s12864-018-4985-2, PMID: 30092779 PMC6085620

[ref53] Silke A. Baldwin I. T. (2010). Insects betray themselves in nature to predators by rapid isomerization of green leaf volatiles. Science 329, 1075–1078. doi: 10.1126/science.1191634, PMID: 20798319

[ref54] Sudhir K. Glen S. Li M. Christina K. Koichiro T. (2018). MEGA X: molecular evolutionary genetics analysis across computing platforms. Mol. Biol. Evol. 35, 1547–1549. doi: 10.1093/molbev/msy096, PMID: 29722887 PMC5967553

[ref55] Tamara V. Marta M. Miguel Angel L. Jorge V. Tomas C. Liam D.. (2007). Oxylipins produced by the 9-lipoxygenase pathway in *Arabidopsis* regulate lateral root development and defense responses through a specific signaling cascade. Plant Cell 19, 831–846. doi: 10.1105/tpc.106.046052, PMID: 17369372 PMC1867370

[ref56] Timothy B. L. Nadya W. Chris M. Wilfred W. L. (2006). MEME: discovering and analyzing DNA and protein sequence motifs. Nucleic Acids Res. 34, W369–W373. doi: 10.1093/nar/gkl198, PMID: 16845028 PMC1538909

[ref57] Ullah A. Sun H. Hakim X. Yang, and Zhang, X. (2017). A novel cotton WRKY-gene, *GhWRKY6-like*, improves salt tolerance by activating the ABA signalling pathway and scavenging of reactive oxygen species. Physiol. Plant. 162, 439–454. doi: 10.1111/ppl.12651 29027659

[ref58] Umate P. (2011). Genome-wide analysis of lipoxygenase gene family in *Arabidopsis* and rice. Plant Signal. Behav. 6, 335–338. doi: 10.4161/psb.6.3.13546, PMID: 21336026 PMC3142411

[ref59] Upadhyay R. K. Handa A. K. Mattoo A. K. (2019). Transcript abundance patterns of 9- and 13-Lipoxygenase subfamily gene members in response to abiotic stresses (heat, cold, drought or salt) in tomato (*Solanum lycopersicum L*.) highlights member-specific dynamics relevant to Each stress. Genes 10. doi: 10.3390/genes10090683, PMID: 31492025 PMC6771027

[ref60] Veldink G. A. Vliegenthart J. F. Boldingh J. (1977). Plant lipoxygenases. Prog. Chem. Fats Other Lipids 15, 131–166. doi: 10.1016/0079-6832(77)90014-3 195311

[ref61] Wankun D. Yongbo W. Zexian L. Han C. Yu X. (2014). HemI: a toolkit for illustrating heatmaps. PLoS One 9:e111988. doi: 10.1371/journal.pone.0111988, PMID: 25372567 PMC4221433

[ref62] Willis D. (2005). *Aspergillus* infection inhibits the expression of peanut 13S-HPODE-forming seed lipoxygenases. Mol. Plant Microbe Interact. 18, 1081–1089. doi: 10.1094/MPMI-18-1081, PMID: 16255247

[ref63] Yan J. Chia J. C. Sheng H. Jung H. I. Zavodna T. O. Lu Z.. (2017). *Arabidopsis* pollen fertility requires the transcription factors CIT1 and SPL7 that regulate copper delivery to anthers and Jasmonic acid synthesis. Plant Cell 29, 3012–3029. doi: 10.1105/tpc.17.00363, PMID: 29114014 PMC5757271

[ref64] Ying Z. A. Yz B. (2020). Effect of lipoxygenase-3 on storage characteristics of peanut seeds - ScienceDirect. J. Stored Prod. Res. 87:101589. doi: 10.1016/j.jspr.2020.101589

[ref65] Yu L. H. Wu S. J. Peng Y. S. Liu R. N. Xiang C. B. (2015). *Arabidopsis* EDT1/HDG11 improves drought and salt tolerance in cotton and poplar and increases cotton yield in the field. Plant Biotechnol. J. 14, 72–84. doi: 10.1111/pbi.12358, PMID: 25879154 PMC11389178

[ref66] Zhang J. (2003). Evolution by gene duplication: an update. Trends Ecol. Evol. 18, 292–298. doi: 10.1016/S0169-5347(03)00033-8, PMID: 34850127

[ref67] Zhang Y. Luan Q. Jiang J. Li Y. (2021). Prediction and utilization of malondialdehyde in exotic pine Under drought stress using near-infrared spectroscopy. Frontiers. Plant Sci. 12. doi: 10.3389/fpls.2021.735275, PMID: 34733301 PMC8558207

[ref68] Zhou G. Qi J. Ren N. Cheng J. Erb M. Mao B.. (2009). Silencing *OsHI-LOX* makes rice more susceptible to chewing herbivores, but enhances resistance to a phloem feeder. Plant J. 60, 638–648. doi: 10.1111/j.1365-313X.2009.03988.x, PMID: 19656341

[ref69] Zhuang W. Chen H. Yang M. Wang J. Pandey M. K. Zhang C. (2019). The genome of cultivated peanut provides insight into legume karyotypes, polyploid evolution and crop domestication. Nat. Genet. 51, 865–876. doi: 10.1038/s41588-019-0402-2, PMID: 31043757 PMC7188672

